# The short-chain fatty acid acetate reduces appetite via a central homeostatic mechanism

**DOI:** 10.1038/ncomms4611

**Published:** 2014-04-29

**Authors:** Gary Frost, Michelle L. Sleeth, Meliz Sahuri-Arisoylu, Blanca Lizarbe, Sebastian Cerdan, Leigh Brody, Jelena Anastasovska, Samar Ghourab, Mohammed Hankir, Shuai Zhang, David Carling, Jonathan R. Swann, Glenn Gibson, Alexander Viardot, Douglas Morrison, E Louise Thomas, Jimmy D. Bell

**Affiliations:** 1 Faculty of Medicine, Nutrition and Dietetic Research Group, Division of Diabetes, Endocrinology and Metabolism, Department of Investigative Medicine, Imperial College London, Hammersmith Campus, London W12 0NN, UK; 2 Metabolic and Molecular Imaging Group, MRC Clinical Science Centre, Imperial College London, London W12 0NN, UK; 3 Laboratory for Imaging and Spectroscopy by Magnetic Resonance (LISMAR), Instituto de Investigaciones Biomédicas de Madrid ‘Alberto Sols’ C.S.I.C./U.A.M., Madrid 28029, Spain; 4 Cellular Stress Group, MRC Clinical Science Centre, Imperial College London, London W12 0NN, UK; 5 Food Microbial Sciences Unit, Department of Food and Nutritional Sciences, University of Reading, Reading RG6 6AP, UK; 6 Stable Isotope Biochemistry Laboratory, Scottish Universities Environmental Research Centre, Rankine Avenue, Glasgow G75 0QF, UK

## Abstract

Increased intake of dietary carbohydrate that is fermented in the colon by the microbiota has been reported to decrease body weight, although the mechanism remains unclear. Here we use *in vivo*^11^C-acetate and PET-CT scanning to show that colonic acetate crosses the blood–brain barrier and is taken up by the brain. Intraperitoneal acetate results in appetite suppression and hypothalamic neuronal activation patterning. We also show that acetate administration is associated with activation of acetyl-CoA carboxylase and changes in the expression profiles of regulatory neuropeptides that favour appetite suppression. Furthermore, we demonstrate through ^13^C high-resolution magic-angle-spinning that ^13^C acetate from fermentation of ^13^C-labelled carbohydrate in the colon increases hypothalamic ^13^C acetate above baseline levels. Hypothalamic ^13^C acetate regionally increases the ^13^C labelling of the glutamate–glutamine and GABA neuroglial cycles, with hypothalamic ^13^C lactate reaching higher levels than the ‘remaining brain’. These observations suggest that acetate has a direct role in central appetite regulation.

## Abstract (web summary)

The consumption of fermentable carbohydrates, or fibre, is associated with weight loss. Here the authors show that the metabolite acetate, created by fermentation of fibre in the mouse colon, is taken up into the brain where it induces appetite-suppressing neuronal activity in the hypothalamus.

## Graphical abstract

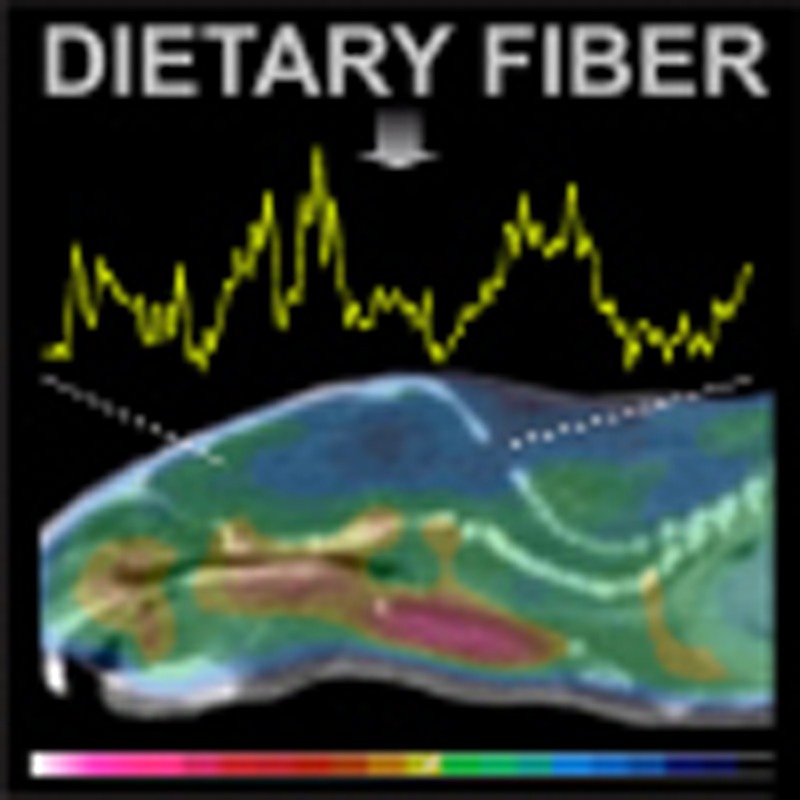

Obesity has reached epidemic proportions worldwide, with incidence rates above 20% in most western countries now representing a major public health burden^1^. This rise in obesity is fuelled by the mismatch between an inherited genetic predisposition for survival in environments where food supply is limited and the current obesogenic environment where reduced physical activity and excess energy intake prevails^2^. Modern food processing has resulted in the mass production of cheap, energy-dense foods that are generally high in refined sugars and fats but low in fibre^3^. It has been estimated that the Palaeolithic diet delivered >100 g per day of fibre, whereas current western intakes are only between 10 and 20 g per day^4^. Much of the fibre consumed in the Palaeolithic diet, unlike current fibre intake, would have been highly fermentable. There is a growing body of evidence that links fermentation of dietary carbohydrate (including fibre) by the colonic microbiota to positive effects on metabolism^5^. Observations by Cani *et al.*^6^ demonstrated that rats exposed to a high-fat diet (HFD) with an increased fermentable carbohydrate (FC) component for 4 weeks had lower dietary energy intake, body weight and adiposity, suggesting that increased dietary FC leads to improved appetite and body weight regulation. FCs are thought to promote the release of the anorectic gut hormones peptide YY (PYY) and glucagon-like peptide-1 (GLP-1) (ref. 7). However, FC-associated weight loss in human and rodent models have failed to consistently show increases in these anorectic hormones^8^.

We have recently shown in mice that dietary FC supplementation is associated with reduced energy intake, body weight, adiposity and changes in hypothalamic neuronal activation patterns, all of which were independent of changes in circulating concentrations of GLP-1 (ref. 9). To better understand the mechanism behind the anorectic action of FC supplementation, we investigated the direct effects of the most abundant end product of colonic FC fermentation, the short-chain fatty acid (SCFA) acetate, on the control of appetite.

We present herein that acetate derived from the colonic fermentation of FC acts to directly supress appetite through central hypothalamic mechanisms involving changes in transcellular neurotransmitter cycles. This, therefore, opens up new research directions into the promotion of acetate production by colonic microbiota and therapeutic strategies for the prevention and treatment of obesity.

## Results

### The effect of FCs on body weight

**Figure 1:**
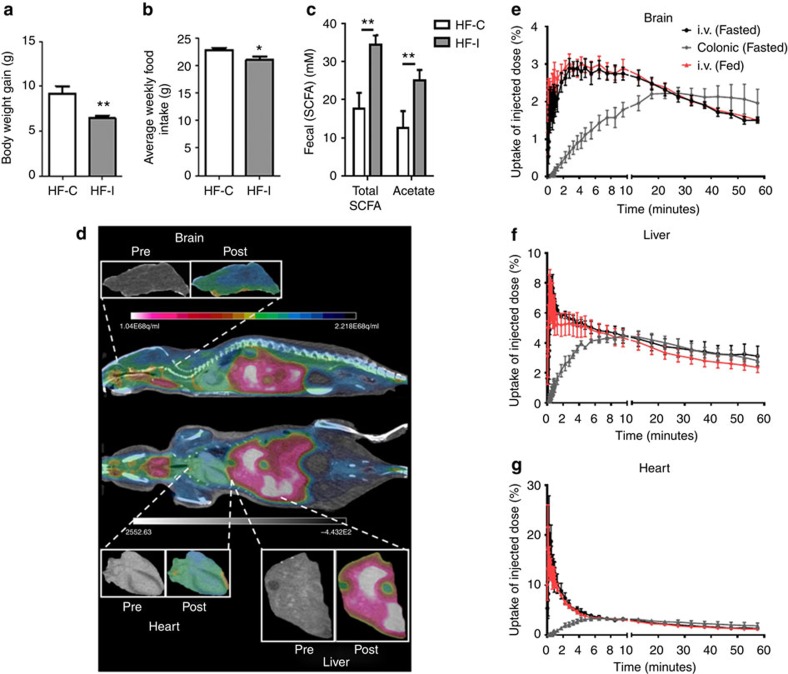
FCs reduce food intake and increase faecal SCFA concentrations. (**a**) Body weight gain and (**b**) average weekly food intake of mice fed with either a HFD supplemented with the highly fermentable fibre inulin (HF-I) or a relatively non-fermentable fibre cellulose (HF-C). Body weight gain and average food intake significantly reduced in the HF-I group, **P<0.01, **P<0.05 based on two-sided, unpaired Student’s *t*-test(*n*=12 per group). (**c**) Total and acetate-only faecal SCFA concentrations obtained from HF-C and HF-I fed mice **P<0.01 based on two-sided, unpaired Student’s *t*-test (*n*=6 per group randomly selected from the *n*=24 cohort). (**d**) The biodistribution of carbon-11 (^11^C) i.v. acetate as determined using PET scanning. Image depicts a fasted mouse following i.v. infusion. Image shows uptake in the brain, liver and heart. (**e**–**g**) Brain, liver and heart uptake of i.v. and colon infused ^11^C-acetate as expressed as a percentage of the initial dose administered. No significant differences, when compared by GEE and Mann–Whitney *U*-test, were observed between i.v. administrations in the fed or fasted state, but there is a slower increase when the ^11^C-acetate was colonically infused (*P*<0.001; *n*=3–4 per group).

We fed C57BL/6 mice with HFD supplemented with either the FC inulin (HF-I) or the poorly fermentable fibre cellulose (HF-C). After 8 weeks, mice fed the HF-I diet gained significantly less weight than those fed HF-C (Fig. 1a) and consumed significantly less food (Fig. 1b). SCFA analysis of the colonic content of the former animals demonstrated a significant increase in total SCFA concentrations and especially the most abundant SCFA, acetate (Fig. 1c). Although others have reported that FC supplementation leads to an increase in the anorectic hormones GLP-1 and PYY^6^, our group has recently reported that FC-rich diets increase manganese-enhanced MRI (MEMRI) signal in the hypothalamus, a proxy of neuronal activation, via a mechanism that appears to be independent of changes in GLP-1 (ref. 9). In support of this, infusion of anorectic gut hormones have previously been shown to suppress hypothalamic MEMRI signal^10^, thereby suggesting that any increase in signal seen following FC supplementation is not PYY or GLP-1 dependent. We therefore hypothesized that there was a direct effect of SCFAs on appetite regulation, namely by acetate, given it is the most abundant SCFA produced in the colon, and that it partially avoids hepatic clearance to reach micromolar concentrations in the peripheral circulation.

### The biodistribution of acetate and uptake in the brain

SCFA have traditionally been thought to be almost entirely metabolized by colonocytes and the liver^11^. However, acetate is present in the peripheral circulation, in human cerebrospinal fluid^12^ as well as in the brain where it provides an important energy source for glial cells^13^. To our knowledge, there has been no previous publication investigating acetate as a potential appetite-regulating agent. We therefore sought to investigate the biodistribution of acetate administered peripherally or by administering it colonically to mimic the endogenous production from FC. To do this, we used *in vivo* intravenous (i.v.) and colonic infusions of ^11^C-acetate and PET-CT in mice. A representative PET-CT image of a fasted mouse following i.v. ^11^C-acetate infusion is shown in Fig. 1d. The liver and heart demonstrated the greatest proportion of initial ^11^C-acetate dose uptake (Fig. 1f,g), but our data also show that up to 3% of the initial acetate tracer was taken up by the brain following i.v. infusions in both the fed and fasted states (Fig. 1e). Brain uptake following colonic infusion was more gradual, but it reached an equivalent level after ~20 min.

### Acetate reduces acute food intake

**Figure 2:**
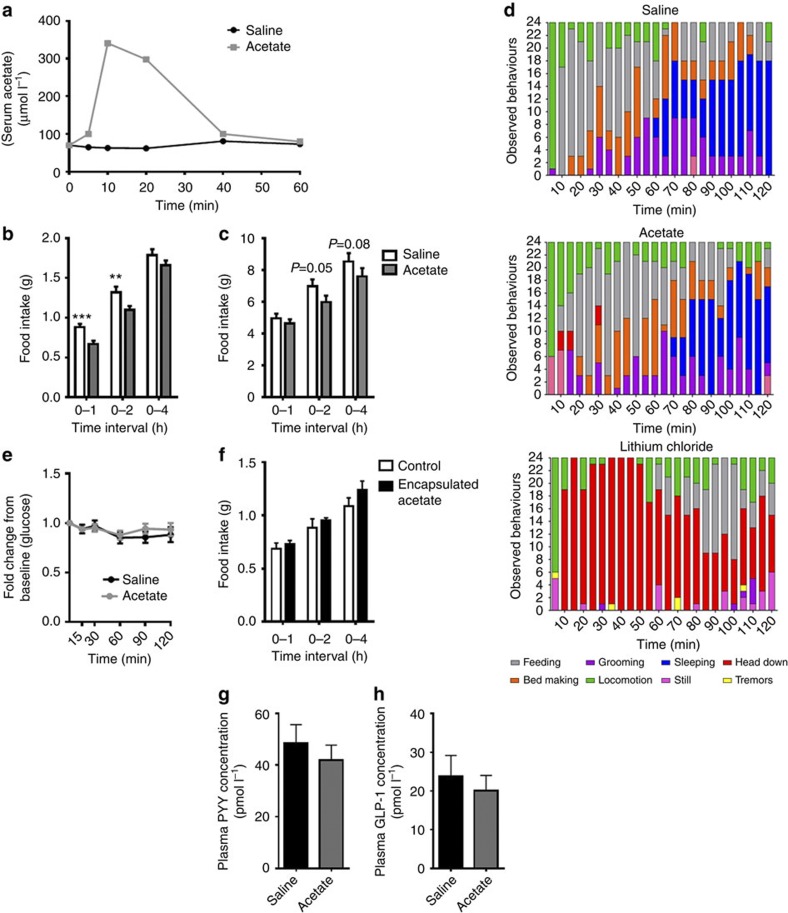
Acetate administration reduces food intake. (**a**) Serum acetate concentrations of mice administered with 500 mg kg^−1^ i.p. acetate or saline (*n*=12). (**b**) Acute food intake data showing food intake in mice following the acute i.p. administration of acetate (500 mg kg^−1^) or saline. Food Intake significantly reduced at 0–1 and 0–2 h; ***P<0.001, **P<0.01 (*n*=21–22 per group). (**c**) The effect of 2.5 μmol of sodium acetate administered into the third ventricle of cannulated rats on food intake compared with a sodium chloride control injection. Cross-over data (*n*=15) compared using two-sided, paired Student’s *t*-test (*P*=0.05, *P*=0.08). (**d**) Behavioural response observed in mice administered either i.p. acetate, saline or lithium chloride (positive control). There was no significant effect on behaviour between saline or acetate as compared using Kruskal–Wallis non-parametric ANOVA (*n*=8). (**e**) Change in baseline blood glucose concentrations (fold change) following 500 mg kg^−1^ sodium acetate or saline injection in *ad libitum* fed mice (treatment effect *P*=0.6 as determined by two-way ANOVA; *n*=9–10 per group). (**f**) The effect of i.p. liposome-encapsulated acetate on acute food intake. No significant difference throughout based on two-sided, unpaired Student’s *t*-test (*n*=7). (**g**,**h**) Effects of i.p. acetate on plasma concentrations of appetite-regulating gut peptides PYY and GLP-1. Measurements made 60 min post injection. There was no significant effect on either peptide as determined by two-sided, unpaired, Student’s *t*-test.

We went on to test whether acetate was itself an anorectic signal using acute peripheral administration in C57BL/6 mice. Intraperitoneal (i.p.) injection of acetate (500 mg kg^−1^) produced an increase in serum acetate (Fig. 2a), similar to those that have been associated with suppression of free fatty acids^14^, which was associated with a significant reduction in acute food intake at both 1 and 2 h post injection (Fig. 2b). An acetate tolerance test demonstrated that there was no impact of i.p. acetate at 500 mg kg^−1^ body weight on blood glucose concentrations (Fig. 2e). Acute behavioural observations post injection showed that this was also not associated with aversive behaviour (Fig. 2d). Furthermore, at 60 min post injection, there were no significant differences in circulating concentrations of plasma PYY and GLP-1 (Fig. 2g,h). We encapsulated acetate into liposomes^15^. Using this method of peripheral administration of liposome-encapsulated acetate over a 4-h period, we did not detect liposomes in the hypothalamus or changes in food intake (Fig. 2f). We therefore hypothesized that the anorectic effects of acetate may result from a direct effect on the central nervous system. We tested this hypothesis by administering 2.5 μmol of sodium acetate directly into the third ventricle of intracerebroventricular (i.c.v.) cannulated male Wistar rats. In accordance with the anorectic effects of peripherally administered acetate, central administration of acetate into the third ventricle suppressed food intake primarily at 1–2 h post injection, resulting in reduced cumulative food intake at both at 2 and 4 h (Fig. 2c), although this effect was not as potent as the suppression observed after i.p. administration of acetate in mice. The short-term suppressions of feeding were as expected given the rapid hepatic metabolism and short half-life of acetate.

### Acetate induces an anorectic neuropeptide expression profile

**Figure 3:**
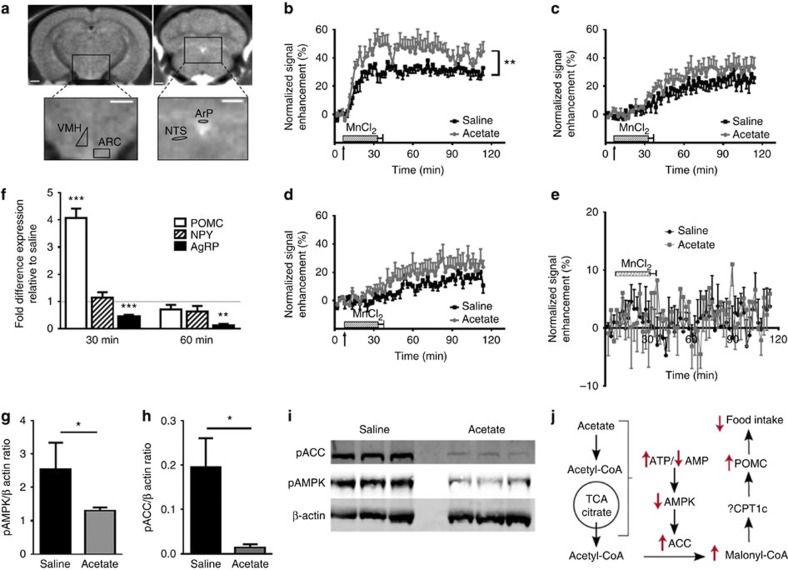
Acetate effects in the hypothalamus. (**a**) Regions of interest (ROIs) used in manganese-enhanced MRI (MEMRI). MR image showing the ROI locations in the hypothalamus from which signal intensity (SI) measurements were determined. ARC, arcuate nucleus; VMH, ventromedial hypothalamus; ArP, area postrema; NTS, nucleus of solitary tract. White bar represents 1 mm. (**b**–**e**) Hypothalamic neuronal activation in the ARC, VMH, the PVN and the NTS of mice following i.p. administration of acetate or a saline control as determined by MEMRI. Arrow signifies start of i.v. MnCl_2_infusion. SI is significantly increased in the ARC of acetate treated mice compared with saline-injected controls based on generalized estimated equations (GEE) and Mann–Whitney *U*-test; **P<0.01 (*n*=4–5 per group). (**f**) Effect of acetate on hypothalamic expression of POMC, NPY and AgRP as determined by hypothalamic qPCR. Data expressed as fold change in expression compared with saline-injected controls at 30 and 60 min post administration one-way ANOVA with *post hoc* Dunnett’s correction; **P<0.01, ***P<0.001 (*n*=5 per group). (**g**,**h**) pACC and pAMPK hypothalami content expressed in relation to β-actin control, based on two-sided, unpaired Student’s *t*-test; *P<0.05 (*n*=5). (**i**) Immunoblots of hypothalamic pAMPK and pACC levels in mice 30 min after the i.p. injection of either saline or acetate (full blot with annotation available in Supplementary Fig. 1). (**j**) Proposed mechanism of acetate induced inhibition of the feeding impulse. Relatively increased hypothalamic TCA cycle activity increases ATP production, decreases AMP levels and AMPK inhibition of acetyl-CoA carboxylase (ACC), increases malonyl-CoA levels and stimulates POMC mRNA expression, and inhibits appetite.

Having confirmed that acetate was an anorectic agent and was indeed taken up by the brain following colonic or i.v. administration, we next sought to confirm whether changes in hypothalamic neuronal activation that we had previously observed following dietary supplementation of FC^9^ could be replicated following an acute administration of acetate. To achieve this, we again used the *in vivo* functional imaging technique MEMRI^9^,^10^,^16^. The regions of interest examined were as shown in Fig. 3a. Mice given an i.p. injection of an anorectic dose of acetate had a significant increase in signal intensity in the arcuate nucleus (ARC), indicative of increased neuronal activation, whereas no significant differences were observed in the ventral medial hypothalamus (VMH), the paraventricular nucleus (PVN) (Fig. 3b–d) or the brain stem (Fig. 3e). These data show an identical pattern to those seen when we fed mice a HFD supplemented with FC inulin^9^. We went on to investigate specific changes in hypothalamic neuropeptide expression following i.p. acetate administration. There was a four-fold rise in the expression of the melanocortin precursor pro-opiomelanocortin (POMC) and a potent suppression of agouti-related peptide (AgRP) expression 30 min after acetate administration (Fig. 3f). At 60 min, this suppression of AgRP remained. This disparate change in the expression patterns of anorectic and orexigenic neuropeptides favour a reduction in appetite and body weight, and have been previously reported by others feeding rodents FC-rich diets^17^.

### Acetate reduces hypothalamic AMPK catalytic activity

Mammalian neuronal tissue is thought to rapidly convert acetate to acetyl-CoA, which then enters the Krebs cycle^13^. Accumulating evidence suggests that hypothalamic AMP-activated protein kinase (AMPK) has an important role in energy and nutrient sensing^18^. Phosphorylation of threonine 172 (T172) within the alpha subunit of AMPK directly activates the kinase, which in turn phosphorylates and inactivates acetyl-CoA carboxylase (ACC), leading to decreased levels of malonyl-CoA. Studies have shown that increased hypothalamic malonyl-CoA concentrations are associated with an increase in the expression of POMC^19^ and suppression of Neuropeptide Y (NPY) and AgRP with a subsequent reduction in rodent food intake. Thus, we hypothesized that acetate administration may mediate the changes in appetite we had observed and also more specifically changes in POMC and AgRP expression. We measured the phosphorylation of key residues of AMPK (T172) and ACC (S79) in mouse hypothalamic lysates following i.p. acetate injection as a surrogate of their activities. We found a significant reduction in levels of pT172 of AMPK and pS79 of ACC (Fig. 3g–i), suggesting that acute acetate administration inactivated AMPK, thereby leading to increased ACC activity. Increased ACC activity has been shown to elevate malonyl-CoA that can stimulate expression of POMC and cocaine- and amphetamine-regulated transcript and decrease NPY and AgRP leading to a reduction in food intake^19^. This therefore suggests that the anorectic effects of acetate administration are mediated through a change in hypothalamic ACC and AMPK activities and downstream changes in neuropeptide expression. A schematic illustration of this relationship is shown in Fig. 3j.

### ^13^C acetate preferentially accumulates in the hypothalamus

**Figure 4:**
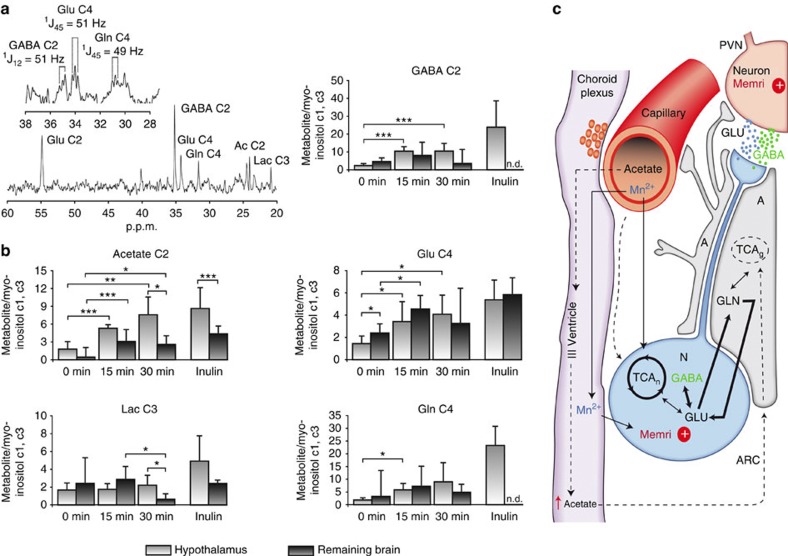
Effects of peripheral (2-^13^C) acetate or intragastric (U-^13^C) inulin administrations on hypothalamic and cerebral metabolism. (**a**) Representative ^13^C (125.03 MHz) HR-MAS spectra (4 °C, 5 kHz) of the hypothalamus from a mouse fasted overnight, 15 min after i.p. [2-^13^C] acetate administration (500 mg kg^−1^). Inset: Representative ^13^C HR-MAS spectra (28–38 p.p.m.) from the hypothalamus of an overnight-fasted mouse, 180 min after [U-^13^C] inulin administration (100 mg) by gavage. (**b**) Increases in ^13^C incorporation into the acetate C2, GABA C2, Glu C4, Gln C4 and Lac C3 carbons (mean+s.d.) in the hypothalamus and remaining brain biopsies, following 0, 15, 30 min i.p. [2-^13^C] acetate (n=18) or 180 min intragastric [U-^13^C] inulin administrations *P<0.05, **P<0.01, ***P<0.001 (n=4). (**c**) Summary of the effects of [2-^13^C] acetate or [U-^13^C] inulin administrations in hypothalamic metabolism. Extracellular [2-^13^C] or ^13^C_2_ acetate derived from plasma or cerebrospinal fluid are transported primarily to the astrocytes, where they are metabolized oxidatively in the TCA cycle, labelling astrocytic glutamate C4 and glutamine C4 that exchange with the corresponding neuronal pools through the glutamate–glutamine cycle, labelling GABA only in gabaergic neurons. (2-^13^C) or ^13^C_2_ acetate are also oxidatively recycled in the neuronal cycle, originating the Lac C3 resonance. The glutamate–glutamine and GABA cycles support glutamatergic and gabaergic neurotransmissions, the two fundamental synaptic events triggering increased Ca^2+^ uptake and competitive Mn^2+^ uptake (MEMRI) that determine the appetite impulse.

Given the aforementioned changes in neuronal activation and neuropeptides expression, we sought to determine the hypothalamic metabolism of acetate using ^13^C high-resolution magic-angle-spinning (HR-MAS). Figure 4 summarizes the results on hypothalamic metabolism of i.p. (2-^13^C) acetate or ^13^C_2_ acetate derived from colonic fermentation of (U-^13^C) inulin administered by gavage. ^1^H-decoupled ^13^C HR-MAS spectra of cerebral biopsies provide information on the operation of the tricarboxylic acid cycle (TCA) and the transcellular glutamate–glutamine and γ-amino butyric acid (GABA) cycles^20^,^21^. Here we implemented the ^13^C HR-MAS approach to investigate the metabolism of labelled [2-^13^C] acetate or ^13^C_2_ acetate in the hypothalamus of fasted mice as well as in biopsies from the ‘remaining brain’ obtained for comparison. To this end, brain metabolism was arrested with focused microwaves immediately after (0 min) or 15 and 30 min later, following the i.p. administration of (2-^13^C) acetate or 180 min after intragastric (U-^13^C) inulin administrations, and a biopsy of the hypothalamus surgically separated from the remaining brain, investigating ^13^C labelling in both samples. Figure 4a depicts ^13^C HR-MAS spectra from a representative mouse hypothalamus 15 min after [2-^13^C] acetate administration, revealing extensive ^13^C incorporation and indicating the most relevant resonances observed. The inset shows a representative ^13^C HR-MAS spectrum (28–38 p.p.m.), obtained from the hypothalamus of a mouse, 180 min after (U-^13^C) inulin administration by gavage. The presence of clearly resolved doublets in hypothalamic glutamate C4 (^1^J_45_=51 Hz), glutamine C4 (^1^J_45_=49 Hz) and GABA C2 (^1^J_12_=51 Hz) demonstrates that ^13^C_2_ acetate units produced by colonic fermentation of (U-^13^C) inulin have been incorporated into hypothalamic metabolism. Figure 4b shows the time course of ^13^C incorporation from these substrates in acetate C2, glutamate and glutamine C4, GABA C2 and lactate C3 carbons in the hypothalamus biopsy (light grey), as well as in the remaining brain biopsy (dark grey). The ^13^C HR-MAS resonance of acetate C2 increased significantly with time reaching higher intensity in the hypothalamus than in the remaining brain, revealing a heterogeneous distribution of cerebral acetate levels with a preferential accumulation of this substrate in the hypothalamus. In addition, the ^13^C resonances of Glu C4, Gln C4 and GABA C2 also increased significantly with time both in intra- and extra-hypothalamic structures, supporting substantial metabolism of accumulated ^13^C acetate in the cerebral TCA cycle and neuroglial glutamate–glutamine or GABA transcellular cycles (Fig. 4c). To investigate whether the concentrations of acetate derived from fermentation in the colon would be sufficiently high to unambiguously label these metabolites in the hypothalamus (and trigger the anorectic responses described), we used (U-^13^C) inulin. Interestingly, ^13^C accumulation in these metabolites from intragastric (U-^13^C) inulin was often higher than from i.p. (2-^13^C) acetate, revealing an efficient fermentation of the inulin in the mouse colon at a level that directly influences hypothalamic metabolism.

^13^C_2_ acetate from U-^13^C inulin labelled faster Glu C4, Gln C4 and GABA C2 in the hypothalamus than in the rest of the brain. In fact, no detectable ^13^C incorporations in GABA C2 or Gln C4 could be found in the remaining brain biopsies, even when ^13^C incorporation into the common Glu C4 precursor was similar in both regions. We believe that this is the result of the preferential incorporation of ^13^C_2_ acetate and its metabolism in the hypothalamus as compared with the remaining brain, suggesting that Gln C4 and GABA C2 labelling in non-hypothalamic structures would occur at later times.

Notably, ^13^C GABA and ^13^C lactate remained at a similar level until 30 min when both ^13^C GABA and ^13^C lactate were higher in the hypothalamus than in the remaining brain biopsies, indicating a relative increase in gabaergic neurotransmission and oxidative ^13^C lactate production from the pyruvate recycling pathway in the mouse hypothalamus under appetite stimulation^22^.

**Figure 5:**
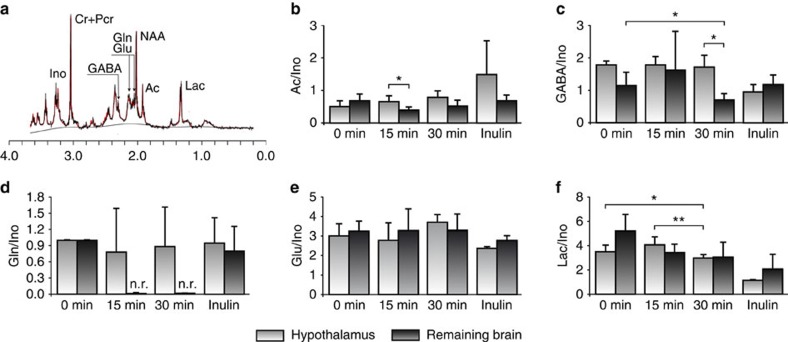
Relative changes in total metabolite content in the hypothalamus and remaining brain biopsies after (2-^13^C) acetate or (U-^13^C) inulin administrations. (**a**) ^1^HR-MAS spectrum from a representative biopsy from the hypothalamus (black) and superimposed LCModel fitting (red). (**b**–**f**) Relative ^1^H HR-MAS ratios Ac/Ino, GABA/Ino, Glu/Ino, Gln/Ino and Lac/Ino at increasing times after (2-^13^C) acetate or (U-^13^C) inulin administrations. Lac, lactate; Ac, acetate;, Glu, glutamate; Gln, glutamine; GABA, γ-amino butyric acid; Cr+PCr, creatine+phosphocreatine; Ino, *myo*-inositol; n.r, not resolved; CRLB, Cranmer–Rao Lower Bound <20%. Bar graphs represent the mean and standard deviation. Statistical significance was evaluated using two-tailed unpaired Student t-test. “Remaining brain” accounts for cerebral extra-hypothalamic tissue. *P<0.05, **P<0.01.

In order to evaluate the contribution of potential variations in total metabolite content to the ^13^C incorporation profiles, we acquired ^1^H HR-MAS spectra for the same biopsies we used for the ^13^C HR-MAS acquisitions, as the resonances detected by ^1^H HR-MAS are known to reveal the total metabolite content. Figure 5a, shows a representative ^1^H HR-MAS spectrum from a biopsy obtained from the hypothalamus, 180 min after (U-^13^C) inulin administration (black trace), with some of the most relevant metabolite resonances highlighted and the corresponding LCModel fitting superimposed (red trace). We calculated the metabolite ratios for acetate, GABA, glutamate, glutamine and lactate using the total *myo*-inositol or the total creatine (Cr+PCr) resonances as internal references. Our results revealed that the significantly higher ^13^C incorporations in hypothalamic acetate C2, GABA C2, glutamate and glutamine C4 and Lac C3 are primarily derived from the ^13^C-labelled substrates administered rather than from relative changes in total metabolite content (Fig. 5b–f). Notably, similar levels of total glutamine and total GABA were found in the hypothalamus and remaining brain biopsies 180 min after (U-^13^C) inulin administration (Fig. 5c,d). confirming that the higher ^13^C incorporations detected in hypothalamic Gln C4 and GABA C2, are not derived from relative changes in the total metabolite levels between these two regions. Similar conclusions were obtained when using the total creatine (Cr+PCr) resonance as internal reference for the total metabolite content. This normalization allowed, however, for the comparison between the myo-inositol content in the hypothalamus and the remaining brain. No significant differences in Ino/(Cr+PCr) ratios were found between both regions, 15 or 30 min after (2-^13^C) acetate, or 180 min after (U-^13^C) inulin administrations. These results suggest that previously reported differences in myo-inositol content between the untreated mouse hypothalamus and hipoccampus^23^ do not contribute appreciably to the present comparisons between the hypothalamus and remaining brain biopsies after (2-^13^C) acetate or (U-^13^C) inulin administrations.

Summarizing, we conclude then that the neuronal activation in the ARC observed in Fig. 3b using MEMRI occurs because of a relative increase in acetate and glutamatergic or gabaergic neurotransmissions in the hypothalamus that would result in divalent ion uptake, in this case Mn^2+^, and therefore an increase in MEMRI signal intensity as shown in our summary schematic (Fig. 4c).

## Discussion

In this paper, we provide a novel insight into the mechanism behind the well-established observation that animals fed a HFD supplemented with FC have decreased energy intake and weight gain^24^,^25^. Most of the work in this area investigating the mechanism underlying these observations has previously focused on the potential effect of FC to promote the release of the anorectic gut hormones PYY and GLP-1 (ref. 6). However, increased concentrations of these hormones have been difficult to reproduce in humans^26^. In previous studies from our group, we have observed that decreased food intake and diminished body weight can occur independently of change in gut hormones in mice^9^,^24^. We also observed an increase in MEMRI signal in the hypothalamic ARC, with no associated increase in signal intensity in the brain stem, the opposite to that observed following administration of the anorectic gut hormones GLP-1 and PYY^27^. Here we have demonstrated that the decrease in body weight observed in FC supplementation studies appears to be mediated, at least in part, by the SCFA acetate. Acute i.p. injection of acetate decreased energy intake over 2 h without effecting behaviour and occurred independently of changes in the peripheral concentrations of GLP-1 and PYY. It is of interest that encapsulation of acetate into liposomes, which specifically allows for peripheral delivery, abolishes the anorectic effect of acetate in mice, whereas i.c.v. administration of acetate in rats, although still anorectic, shows a milder and more delayed suppression of food intake. The lack of brain-stem-induced changes in signal intensity following i.p. acetate do not suggest a vagally mediated effect of acetate, and thus the difference in potency may be because of species variation. We also demonstrate that appetite changes are unlikely to be because of change in the availability of glucose to the hypothalamus, as acetate did not affect circulating glucose concentrations. We have also shown that acetate administered through both i.v. and colonic routes is taken up by the brain and for the first time that acetate derived from fermentation of carbohydrate in the colon is taken up by the hypothalamus in greater amounts than other brain tissue. MEMRI signal intensity patterning following acetate infusion also directly reflects that of animals fed with FC^9^. Furthermore, we have shown that peripheral acetate administration leads to increased POMC and reduced AgRP expression in the hypothalamus, suggesting that acetate has a direct effect on the hypothalamic control of appetite. Similar observations have been made when animals are fed with FC^17^.

Changes in the phosphorylation of key residues of AMPK and ACC in mouse hypothalamic lysates following peripheral acetate administration also provides evidence of a potential mechanism through which the changes in neuropeptide expression might occur. Given that acetate is taken up by the hypothalamus, where it is preferentially metabolized by astrocytes because of the higher activity of their monocarboxylate transporters compared with neurons^28^,^29^, a non-neuronal mechanism may also be implicated in the observed increase in ARC activation. Using ^13^C HR-MAS in whole hypothalamic tissue, we were able to demonstrate that ^13^C acetate from both i.p. injections and derived from colonic fermentation incorporates into the glutamate–glutamine transcellular cycle, increasing lactate and GABA labelling, thus supporting hypothalamic glutamatergic or gabaergic neurotransmissions. The same model has been proposed to occur in acetate metabolism following alcohol ingestion^30^. The operation of the glutamine cycle involves imperative signalling through ionotropic and metabotropic postsynaptic glutamate receptors, resulting in increased intracellular Ca^2+^ accumulation^22^ and a concomitant MEMRI effect^31^ (Fig. 4c). It has recently been demonstrated that leptin modulates glutamate transporter expression in hypothalamic astrocytes with an associated increase in POMC neuron excitability^32^. A similar mechanism could take place following acetate administration, potentially explaining the increase in MEMRI signal observed in ARC of animals supplemented with FC or given acetate alone. We hypothesize that the increase in hypothalamic ^13^C acetate over time, as compared with rest of the brain, results in increased hypothalamic gabaergic neurotransmission and oxidative lactate production from the pyruvate recycling pathway^22^. Increased oxidative metabolism results in increased ATP production, decreasing the AMP:ATP ratio, and thereby decreasing AMPK activity and consequently reducing the inhibition of ACC. This produces an increase in malonyl-CoA, stimulates POMC neurons and predominantly gabaergic neurotransmission, thus decreasing the appetite impulse^33^.

Dietary intakes of fibre in excess of 100 g per day consumed by Palaeolithic man and in some hunter gather tribes^4^ suggest the human colon is adapted to large intakes of fermentable material that would lead to high-peripheral circulating acetate^34^. The fall in fibre intakes or, more specifically, a decline in the intake of carbohydrates that can be readily fermented in the colon are one such factor that seems to be an inverse correlate of obesity prevalence. Although studies have suggested that the inclusion of highly FC diets may be beneficial in terms of regulation of both appetite and body weight, in free-living individuals, compliance to such eating regimens is often low due to gastrointestinal side effects or unpalatability of FC-rich foodstuffs. The work highlighted within the paper suggests that the SCFA acetate may at least in part mediate some of the obesity-protective effects of FC-rich diets directly in the central nervous system, thus suggesting that acetate may be useful as a potential anti-obesity therapeutic. This hypothesis does not rule out that there are other non-specific effects.

In conclusion, here we provide novel insight into a mechanism through which FC may mediate appetite suppression. By exploring the role of the SCFA acetate, a product of fermentation of carbohydrate in the colon, our evidence suggests that acetate derived from the colon induces an anorectic signal in the hypothalamic ARC by supporting the glutamate–glutamine transcellular cycle and leading to an increase in lactate and GABA production. These data demonstrate a previously unexplored central mechanism through which the fermentation products of FC and dietary fibre may aid in the control of body weight beyond that of energy dilution and gut hormone release. Moreover, it opens up important new possibilities for weight management as the supply of fermentable substrate to the colon (and therefore acetate production) can be modified.

## Methods

### Animals and treatments

Animal experiments were performed at Imperial College London except for high resolution magic angle spinning (HMRS) experiments which were performed at Instituto de Investigaciones Biomédicas ‘Alberto Sols’ (IIB). Experiments conducted at Imperial College London were approved by Imperial College London, and all animal procedures were performed in accordance with the UK Animals Scientific Procedures Act (1986). IIB experiments were approved by the ethical committee of the Instituto de Investigaciones Biomedicas ‘Alberto Sols’ (and met the guidelines of the national (R.D. 53/2013) and the European Community (2010/62/UE) for care and management of experimental animals. Unless otherwise stated, all experiments were performed in C57BL/6 male mice (6–8 weeks old, Charles River, Margate, UK) that were single-housed under controlled temperature (21–23 ^o^C) and light conditions (12 h light–dark cycle; lights on at 07:00 hours).

### The effect of HF-I versus HF-C on body weight and energy intake

Mice were randomized and assigned to two different groups (*n*=12): HFD with cellulose as a control (HF-C), HFD+oligofructose-enriched inulin (Synergy) (HF-I). Synergy is a fructan-based preparation containing both long- and short-chain fructo-oligosaccharides. Synergy was mixed with the HFD individually in the ratio of 1:9. The two diets were isocaloric, each contained the same total energy of 4.6 kcal g^−1^, with 41.8% energy from fat. The diets were made isocaloric by the addition of cellulose. Nutritional composition of the diet is shown in Table 1. The diets were fed *ad libitum* for 8 weeks to respective groups of animals. Body weights and food intake were measured three times per week.

### Determination of acetate concentrations in serum and faeces

SCFAs were determined by gas chromatography in the colonic contents and serum of mice that where freshly culled. The method used was adapted from Richardson *et al.*^35^ Briefly, caecal contents were weighed (20–220 μg) and combined with 550 μl of PBS. Samples were vortex mixed for 1 min, centrifuged at 3,000 *g* for 10 min and the supernatant was collected. To 500 μl supernatant, 25 μl of internal standard and 2-ethylbutyric acid was added to give a final concentration of 5 mmol l^−1^. Acids were extracted by the addition of 250 μl concentrated hydrochloric acid and 1 ml diethyl ether followed by vortex mixing for 1 min. Samples were centrifuged for 10 min at 3,000 *g* and the ether layer was removed and transferred to a separate capped vial. *N*-methyl-*N*-t-butyldimethylsilyltrifluoroacetamide (MTBSTFA; Sigma) was added (100 μl) before heating at 80 °C for 20 min.

Gas chromatography was performed on a Hewlett Packard 5890 Series II instrument equipped with a flame ionization detector, split/splitless injector and a 10-m, 0.18 mm ID and 0.20 μm df Rtx-1 (Crossbond 100% dimethyl polysiloxane, Thames Restek UK, Ltd) capillary column. Injector and detector temperatures were 275 ^o^C with the column temperature programmed from 63 °C for 3 min to 190 ^o^C at 10 °C per min. Helium served as the carrier gas (head pressure 135 kPa) and injections (1 μl) were made in the split mode (50:1 split). Peak areas were recorded and all subsequent data manipulation was completed using ChemStation Software (Agilent Technologies, USA). External standards for acetate, propionate, n-butyrate, iso-butyrate, n-valerate and caproate were prepared at concentrations of 25, 12.5, 6.25, 1.25 and 0.625 mM and ethyl butyric acid was used as the internal standard at a concentration of 100 mM. Reported values were normalized according to the weight of original sample used.

### Serum SCFA measurement

A 100–500-μl aliquot of serum was filtered through a 30-kDa micropartition system (Vivaspin RC VS02H22 filters, Sartorius Inc., Mississauga, ON, Canada) by centrifugation at 14,000 *g* at 4 °C for 90 min. An internal standard solution (25 μl) consisting of 100 mM ethylbutyrate and 100 mM formic acid was added to 225 μl of the protein-free filtrate supernatant in a 2-ml Hichrom vial (Agilent Technologies, South Queensferry, UK). To measure SCFA, 1 μl of each sample was injected into a 5,890 Series II GC system (HP, Crawley, UK) fitted with a NukolTM Capilllary Column (30 m × 0.53 mm × 1.0 μm, SUPELCOTM Analytical, UK) and flame ionization detector. The carrier gas, helium, was delivered at a flow rate of 14 ml min^−1^. The head pressure was set at 10 p.s.i. with split injection. Run conditions were: initial temperature 60 °C, 1 min; +20 °C min^−1^ to 145 °C; +4 °C min^−1^ to 200 °C, hold 25 min. Peaks were integrated using Agilent ChemStation software (Agilent Technologies, Oxford, UK) and SCFA content quantified by single-point internal standard method. Peak identity and internal response factors were determined using a 1-mM calibration cocktail including acetate, propionate and butyrate.

### PET-CT analysis of acetate biodistribution

Mice were fasted overnight. Animals received ^11^C-acetate (~20 MBq) at the beginning of PET scan either through i.v. tail vein (*n*=3) or colonically by using tubing placed 1 cm into the rectum (*n*=4). At the time of the scan, animals were anaesthetized with a 2–2% isofluorane–oxygen mix. After the animals were placed in the micro PET/CT scanner (PET/CT (Siemens), a CT scan without contrast was performed, followed by the PET scan and lastly followed by a CT scan where contrast Ultravist 370 (Bayer, AG, Germany) was infused.

During the scan, animals were maintained at 1–1% isofluorane–oxygen mix and body temperature of 37 °C. The CT had an X-ray source of 80 kVp and 500 μA with exposure time 200 ms and isotropic resolution of 103 μm. The scan consisted of three bed positions with a 20% overlap. The CT scan was used for anatomical data and attenuation correction purposes. The PET system consisted of 64 lutetium oxyothosilicate based detectors blocks arranged in four contiguous rings, with a crystal ring diameter of 16.1 cm and an axial extent of 12.7 cm. Each detector block was composed of a 20 × 20 array of lutetium oxyothosilicate crystals coupled to a position sensitive photomultiplier tube via a light guide. Each crystal was 10 mm long and had a cross-sectional area of 1.51 × 1.51 mm. The crystal pitch was 1.59 mm in both axial and transverse directions. Inveon Research Workplace version 3 (Siemens) was used for data analysis. Images with matrix size 12 × 128, pixel size 0.776 mm^2^ and slice thickness 0.796 mm were reconstructed from the two-dimensional sinogram by two-dimensional-filtered backprojection using a ramp filter with a nyquist frequency of 0.5. Dynamic framing was used in reconstruction with 20 × 3 s frames, 8 × 30 s frames, 5 × 60 s frames and 10 × 300 s frames. Attenuation correction was also used to correct the image. Reconstructed PET images were then registered onto the CT images. PET signals on the desired region of interest were obtained over the scan period (1 h). Percentage injected dose (% ID per g) was calculated by using the following Equations (1) and (2), where; *λ*=0.693/*T*_1/2_, *T*_1/2_ is the half-life of the radionuclide, *I*_t_ is activity at a given time where *I*_0_ is the starting activity and t is the time passed, C_PET_ is the contrast of the image and ID is the activity of the injected acetate.


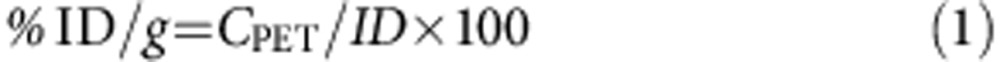



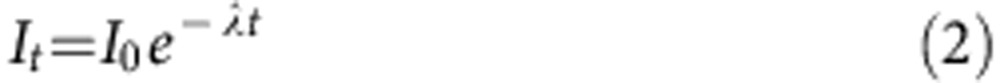


### Effect of i.p. acetate on energy intake

Mice were fasted overnight before receiving either 500 mg kg^−1^ sodium acetate dissolved in 0.9% saline (*n*=22) or vehicle control (0.9% saline; *n*=21) pH 7.0, This dosage of acetate was similar to that used to suppress lipolysis in previous studies and was shown to be well tolerated^14^. Food intake was measured 1, 2, and 4 h post injection.

### Effect of i.c.v. acetate on energy intake

Male Wistar rats 190–240 g (specific pathogen free; Charles River) were placed in a stereotaxic frame (David Kopf Instruments) under 0.5-2.5% isoflurane anaesthesia. A hole was drilled using a stereotactically mounted electric drill using coordinates calculated from the Rat Brain Atlas as described previously^36^, according to the coordinates of Paxinos and Watson^37^ (0.8 mm posterior to the Bregma in the midline and 6.5 mm below the surface of the skull). A permanent 22-gauge stainless steel cannula (Plastics One Inc., Roanoke, Virginia, USA) projecting to the third cerebral ventricle was implanted. Dental cement was used to hold the cannula in position anchored by three stainless steel screws inserted into the skull. The skin was approximated using nylon sutures. After a 7 day recovery period, animals were handled daily and acclimatized to the injection procedure. To ensure that cannulae were correctly positioned, rats received i.c.v. 150 ng angiotensin II in a 5-μl volume via a 28-gauge stainless steel injector placed into and projecting 1 mm below the tip of the cannula. Rats were observed for a prompt drinking response. A total volume of 5 μl was injected over 1 min to conscious, freely moving rats. For feeding studies, rats (*n*=15) were fasted overnight and then injected between 09:00 and 10:00 hours with sodium acetate (2.5 μmol) or an equivalent osmotic sodium chloride control (5 μl) and returned to their home cage. Food intake was measured at 1, 2 and 4 h post injection. After a 72-h washout period, rats received either the acetate or sodium chloride in a cross-over manner and food intake measured as previously described.

### Effect of liposome encapsulate acetate on food intake

Nanoparticle design was based upon our previous studies where PEGylated liposomes were formulated for labelling and visualization of cells^38^, or specifically designed for preferential uptake in xenograft tumours^15^. Liposomes were prepared by the thin-film hydration method^39^. Liposomes were prepared with either acetate (1 M, pH 2.3) to form liposome-encapsulated acetate (LITA) nanoparticles or HEPES for use as a control. Particle size was determined using a Malvern Zetasizer (Malvern Instruments, UK). Quantification of acetate encapsulated within LITA nanoparticles was determined using ^1^H nuclear magnetic resonance (NMR) spectroscopy. LITA formulation (200 μl) was scanned with addition of albumin that binds to acetate in free solution reducing the NMR signal for the compound^40^. A liposome-free control solution containing an equivalent concentration of acetate (4 mM) was also scanned with and without the addition of albumin (2 g). LITA solution was scanned following the addition of lactate (5.2 mg sodium lactate) to act as a quantitative control. Spectra were obtained using a Bruker DRX 11.74T NMR spectrometer. Spectra were analysed using MestRe-C NMR spectroscopy software (Santiago de Compostela, Spain).

To assess short-term biodistribution, a liposome formulation containing an additional 0.1% of a rhodamine–lipid complex (DOPE-Rhodamine: 1,2-dioleoyl-sn-glycero-3-phosphoethanolamine-N-(lissamine rhodamine B sulfonyl) was used to determine biodistibution through *ex vivo* histological analysis. Samples were collected 4 h post i.p. injection (*n*=4). No significant accumulation of liposome was observed in the brain of the treated mice despite being present in the liver and heart.

### The effect of acetate on behaviour

Mice were fasted overnight before receiving either a single i.p. injection of saline, 500 mg kg^−1^ acetate or 2.5 M LiCl as a positive control for aversive behaviour^41^ (*n*=8 per group). The behavioural patterns of each mouse were observed for 15 s every 5 min from the time of injection until 1 h post injection. Behaviours was classified using a modified version of a previously published protocol^42^. Briefly, behaviours was classified into 10 different categories: feeding, drinking, locomotion (including rearing and climbing), bed making, burrowing, grooming, still, sleeping, head down (animal in abnormal body posture: back hunched, eyes shut or half-shut and pilo erect—indicative of impaired health status) and tremors.

### Acetate tolerance test

Glucose levels were determined from blood taken from mouse tails using a Glucometer Elite glucometer (Bayer Corp.). Experiments were performed on *ad libitum* fed mice at 14:00 h as previously described for insulin tolerance tests^18^ but instead of insulin, animals were injected i.p. with either 500 mg kg^−1^ sodium acetate or saline control (*n*=9–10). Blood glucose values were then determined at the times indicated. Results were expressed as fold change of initial blood glucose concentration before injections.

### Plasma PYY and GLP-1 hormone analysis

All samples were assayed in duplicate and in a single assay to eliminate inter assay variation. Plasma PYY and GLP-1 were assessed using an established in-house radioimmunoassay as described previously^43^,^44^.

### Hypothalamic quantitative PCR

This was carried out using the methods described by Bewick *et al.*^45^ Quantitative reverse transcriptase PCR (RT-qPCR) was used to study the expression of the different target genes. Total RNA was extracted from the whole hypothalami using TRIZOL (Invitrogen) according to the manufacturer’s protocol. All samples were treated by DNaseI (Invitrogen) before the reverse transcription. First-strand cDNAs were prepared using 1 μg RNA and SuperScriptII Reverse Transcriptase (Invitrogen) in the presence of random hexamer and oligo(dT) primers (Promega, Charbonnières-les-Bains, France). The qPCRs were performed using the Light Cycler Fast Start DNA Master SyBR Green I kit (Roche, Meylan, France) in the presence of specific primer pairs that were selected to amplify small fragments (100–200 bp). PCR products were checked for specificity by measuring their melting temperature. Samples (in duplicate) were quantified by comparison with a standard curve obtained by dilutions of purified-specific cDNAs.

### Measurements of AGRP and POMC mRNA expression

RNA was extracted from dissected hypothalamus using Absolutely RNA microprep kit from Stratagene (La Jolla, CA, USA). The gene transcription for AgRP and POMC in the ARC of the hypothalamus was determined using real-time RT-PCR, and results were expressed as a ratio to the expression of the constitutive gene cyclophilin. The sequences of TaqMan probes and primers for cyclophilin (GenBank accession no. M15933) were: forward primer 5′-CCCACCGTGTTCTTCGACAT; reverse primer 5′-TGCAAACAGCTCGAAGCAGA-3′; and probe 5′-CAAGGGCTCGCCATCAGCCG-3′. For AGRP, they were: forward primer 5′-TTGGCAGAGGTGCTAGATCCA-3′; reverse primer 5′-AGGACTCGTGCAGCCTTACAC-3′; and probe 5′-CGAGTCTCGTTCTCCGCGTCGC-3′. The probe and primers for POMC (assay identification no. Rn00595020_ml) were purchased from Applied Biosystems.

### Manganese-enhanced MRI

MEMRI was performed using a 9.4-Tesla Varian INOVA imaging system (Varian Inc., USA) as described previously^46^. A fast spin-echo multi-slice sequence was applied with the following parameters: TR= 600 ms, TE=10 ms, matrix size= 256 × 192, FOV=25 × 25 mm and average=1 acquiring 46 × 0.4 mm thick slices. An array of 66 acquisitions was set up so that the 46 slices were acquired 66 times throughout the infusion. Normalized percentage enhancement in signal intensity was calculated for the ARC, VMH, PVN, periventricular nucleus (PE) and the nucleus of tractus solitarius^27^,^43^.

### Hypothalamic measurement of AMP kinase

Animals were killed by decapitation, brains were immediately dissected and the hypothalamus was removed and snap-frozen in liquid nitrogen. Frozen tissues (~100 mg) were homogenized in 0.4 ml of ice-cold 50 mm HEPES, pH 7.4, 50 mm sodium fluoride, 5 mm sodium pyrophosphate, 250 mm sucrose, 1 mm EDTA, 1 mm dithiothreitol, 1 mm benzamidine, 1 mm trypsin inhibitor, 0.1% (w/v) phenylmethylsulfonyl fluoride using an UltraTurax homogenizer (3 × 30-s bursts). Insoluble material was removed by centrifugation and the resulting supernatant was used for immunoprecipitation of AMPK and western blot analysis.

### Immunoblotting and antibodies

Samples were boiled in electrophoresis sample buffer and resolved on polyacrylamide gels. Proteins were transferred into PVDF membrane (Immobilon-FL, Millipore) and blocked with PBS containing 5% skimmed milk powder for 1 h. Antibodies were diluted in 5 ml high salt Tween buffer (20 mM Tris, pH 7.4, 500 mM NaCl and 0.5% Tween (v/v)), and incubated with the membrane. Primary antibodies used for immunoblotting are as follows: anti-AMPK-β1/2 (in-house) (dilutions 1:3,000–1:10,000 for blotting), anti-phospho-ACC (S79) (Cell Signalling, cat. no. 3661), anti-phospho-AMPK (PT172) (Cell Signalling, cat. no. 2535) anti-β-actin (Sigma, cat. no. A5316).

Primary antibodies were detected using fluorescently linked secondary antibodies (Alexa Fluor, Invitrogen, goat anti rabbit: A21109 and goat anti mouse: A21058). These were visualized using an Odyssey infrared imager (Li-Cor Biosciences). Quantification of results was performed using Odyssey software and expressed as a ratio of the signal obtained with the phospho-specific antibody relative to the appropriate total antibody. Full-length images of cut immunoblots are shown in Supplementary Fig. 1.

### The effect of acetate on hypothalamic metabolism

[2-^13^C] acetate (500 mg kg^−1^) or [U-^13^C] inulin (100 mg) were administrated to 8–10-week-old C57BL/6J male mice (Charles River, Spain) after an overnight fasting (16 h) either with i.p. (*n*=18) or by gavage (*n*=4), respectively. All mice were anaesthetized with 4% isoflurane (3 l per min, 99% oxygen), and cerebral metabolism was arrested within 1.5 s using a high-power (5 kW) microwave fixation system (TMW-6402C, Muromachi Kikai Co. Ltd, Tokyo, Japan) immediately (0 min), 15 and 30 min after acetate, or 180 min after inulin, administrations. Fixed brains were dissected and hypothalamus and remaining brain biopsies were obtained and maintained frozen (−80 °C) until HR-MAS analyses. ^13^C (125,03 MHz) and ^1^H HR-MAS (500,13 MHz) spectra were acquired at 11.7 T (4 °C, 4,000 Hz rotation) with a Bruker AVANCE wide bore spectrometer equipped with a MAS accessory (Bruker Biospin, Rheinstetten, Germany). ^1^H-decoupled ^13^C HR-MAS spectra were acquired using a pulse-acquire sequence, with WALTZ-16 ^1^H decoupling applied during the acquisition and relaxation delay periods. Conditions were π/4 pulses, 8,192 or 16,384 scans (for [2-^13^C] acetate or [U-^13^C] inulin measurements, respectively), 64 k data points, 5 s recycle delay. The ^13^C spectra were processed with Mestrelab ( http://mestrelab.com/). Chemical shifts of the ^13^C resonances were referred to the acetate C2 resonance (24.5 p.p.m.), and the ^13^C incorporation was normalized to the myo-inositol C1+C3 resonance (73.2 p.p.m.), which provides a useful internal reference from which all ^13^C resonances can be normalized independently of the amount of tissue^20^,^47^, introducing appropriate corrections for nuclear Overhausser enhancement and saturation.

^1^H HR-MAS spectra from the same biopsies used for ^13^C HR-MAS acquisitions were acquired using the Carr-Purcell-Meiboom-Gil sequence. Acquisition parameters were 5 s water pre-saturation, echo time=144 ms, *t*=1 ms, *n*=100, 10 kHz spectral width, 32 k data points and 256 scans. Metabolite concentrations were evaluated using a modified version of the LCModel processing software (Linear Combination of Model Spectra, http://s-provencher.com/pages/schtm)^48^.

Statistical tests were performed using two-tailed unpaired Student’s *t*-test between values in the different time points or areas. Observations that fell below the Q1-1.5 × IQR (interquartile range) or above the Q3+1.5 × IQR range (with Q_1_ and Q_3_ representing the upper and lower quartiles values and IQR, the difference between Q_3_ and Q_1_) were considered outliers and were not taken into account for statistical evaluations.

### Data analysis

Analyses were performed using Graph Pad Prism (GraphPad Software, San Diego, CA, USA) or Stata (StataCorp LP, College Station, TX, USA). All data was tested for normality using Shapiro–Wilk test. Non-normally distributed data was log transformed to normalize the distribution. Comparison between groups was carried out by two-sided unpaired Student’s *t*-test for two groups and one-way analysis of variance (ANOVA) with *post hoc* Tukey or Bonferroni correction if there were more than two groups. I.c.v. cross-over acetate injection studies were compared using a two-sided paired Student’s *t*-test. Given the cumulative nature of both MEMRI and PET signal intensity data, differences in signal intensity profile between the regions of interest in all experimental groups were analysed using GEE and the Mann–Whitney *U*-test with commercial statistical software (Stata, version 9.1; StataCorp), which compare profiles for the entirety scan. All results and graphs are expressed as means±s.e.m. Results were considered statistically significant when *P*<0.05, with the significance level indicated as *P<0.05, **P<0.01 and ***P<0.001

## Author contributions

All authors had input into the design of the study and had a role in the execution of the experimental methods and data collection. G.F. and M.L.S. coordinated the writing of the manuscript with all authors commenting.

## Additional information

**How to cite this article:** Frost, G. *et al.* The short-chain fatty acid acetate reduces appetite via a central homeostatic mechanism. *Nat. Commun.* 5:3611 doi: 10.1038/ncomms4611 (2014).

## Supplementary Material

Supplementary Information Supplementary Figure 1

## Figures and Tables

**Table 1:** Nutritional content of the HFD-C and HFD-I diets.

**Diets**	**HF-C**	**HF-I**
Ingredients	g kg^−1^	g kg^−1^
Energy (kcal g^−1^)	4.6	4.6
Casein	195.0	195.0
DL-Methionine	3.0	3.0
Sucrose	342.96	342.96
Corn starch	75.0	75.0
Maltodextrin	75.0	75.0
Anhydrous milk fat	210.0	210.0
Cellulose	110.0	50.0
Mineral mix (AIN-76)	35.0	35.0
Calcium carbonate	4.0	4.0
Vitamin mix (Teklad)	10.0	10.0
Ethoxyquin, antioxidant	0.04	0.04
Inulin	0.0	100.0
β-glucan	0.0	0.0

HF-C, high-fat diet with cellulose as a control; HF-I, HFD+oligofructose-enriched inulin.
