# Supplementary material for: The short-chain fatty acid acetate reduces appetite via a central homeostatic mechanism
Source: Nat Commun. 2014 Apr 29;5:3611. doi: 10.1038/ncomms4611 (PMC4015327; doi:10.1038/ncomms4611)
Supplement: Supplementary Information — Supplementary Figure 1 [file ncomms4611-s1.pdf]

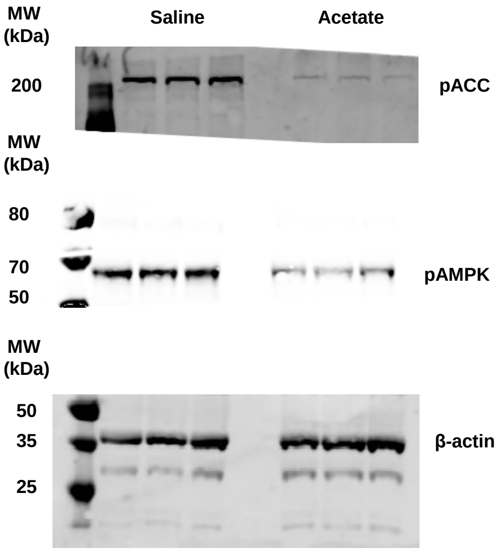

Supplementary Fig. 1:

Full length immunoblots of hypothalamic pAMPK and pACC levels in mice 30 minutes after the intraperitoneal injection of either saline or acetate compared with B-Actin control. Numbers represent molecular weight annotation
